# Essential roles of class E Vps proteins for sorting into multivesicular bodies in *Schizosaccharomyces pombe*

**DOI:** 10.1099/mic.0.2007/006072-0

**Published:** 2007-08

**Authors:** Tomoko Iwaki, Masayuki Onishi, Masaru Ikeuchi, Ayako Kita, Reiko Sugiura, Yuko Giga-Hama, Yasuhisa Fukui, Kaoru Takegawa

**Affiliations:** 1 Department of Life Sciences, Faculty of Agriculture, Kagawa University, Miki-cho, Kagawa 761-0795, Japan; 2 Research Center, Asahi Glass Co. Ltd, Kanagawa, Yokohama 221-8755, Japan; 3 Laboratory of Biological Chemistry, Graduate School of Agricultural and Life Science, University of Tokyo, 1-1-1 Yayoi, Bunkyo-ku, Tokyo 113-8657, Japan; 4 Laboratory of Molecular Pharmacogenomics, School of Pharmaceutical Sciences, Kinki University, Kowakae 3-4-1, Higashi-Osaka 577-8502, Japan

## Abstract

The multivesicular body (MVB) sorting pathway is required for a number of biological processes, including downregulation of cell-surface proteins and protein sorting into the vacuolar lumen. The function of this pathway requires endosomal sorting complexes required for transport (ESCRT) composed of class E vacuolar protein sorting (Vps) proteins in *Saccharomyces cerevisiae*, many of which are conserved in *Schizosaccharomyces pombe*. Of these, *sst4*/*vps27* (homologous to *VPS27*) and *sst6* (similar to *VPS23*) have been identified as suppressors of sterility in *ste12*Δ (*sst*), although their functions have not been uncovered to date. In this report, these two *sst* genes are shown to be required for vacuolar sorting of carboxypeptidase Y (CPY) and an MVB marker, the ubiquitin–GFP–carboxypeptidase S (Ub–GFP–CPS) fusion protein, despite the lack of the ubiquitin E2 variant domain in Sst6p. Disruption mutants of a variety of other class E *vps* homologues also had defects in sorting of CPY and Ub–GFP–CPS. *Sch. pombe* has a mammalian AMSH homologue, *sst2*. Phenotypic analyses suggested that Sst2p is a class E Vps protein. Taken together, these results suggest that sorting into multivesicular bodies is dependent on class E Vps proteins, including Sst2p, in *Sch. pombe*.

## INTRODUCTION

Genetic selections in *Saccharomyces cerevisiae* have resulted in the isolation of a large number of *vps* mutants defective in the delivery of proteins to lysosome-like vacuoles. These define more than 60 complementation groups and have been categorized into six classes (A–F) with respect to their morphology, vacuolar protein sorting and acidification defects (Raymond *et al.*, 1992; Banta *et al.*, 1988). Class E *vps* mutants fail to transport newly synthesized hydrolases efficiently to the vacuole (Raymond *et al.*, 1992; Piper *et al.*, 1995; Rieder *et al.*, 1996). Instead, hydrolases accumulate with endocytosed receptors in an exaggerated perivacuolar class E compartment. In *Sac. cerevisiae* class E *vps* mutants, FM4-64 accumulated asymmetrically at the vacuolar membrane either in the form of a crescent-shape on one side of the vacuole or in the form of a small ring-like structure adjacent to the vacuole (Raymond *et al.*, 1992). Electron microscopic analysis revealed the presence of class E compartments consisting of stacks of curved membrane cisternae in class E *vps* mutants (Rieder *et al.*, 1996; Babst *et al.*, 1997).

The class E *vps* genes are largely conserved in *Schizosaccharomyces pombe* and mammalian genomes (Babst, 2005; Winter & Hauser, 2006; Takegawa *et al.*, 2003), and the majority of the proteins encoded are constituents of three separate heteromeric protein complexes called ESCRT-I, ESCRT-II and ESCRT-III (endosomal sorting complex required for transport) (Babst, 2005; Bowers & Stevens, 2005; Winter & Hauser, 2006). These complexes are proposed to be the sorting machinery for endosomal structures called multivesicular bodies (MVBs). MVBs are part of the endosomal system for degradation of transmembrane proteins, and are formed by invagination and budding of vesicles from the limiting membrane of endosomes into the lumen of the compartment. Two types of MVB cargo proteins are found in budding yeast. One group consists of the endocytosed surface proteins that function as transporters (e.g. Gap1p, Fur4p and Ste6p) and receptors (e.g. Ste2p and Ste3p) (Rieder *et al.*, 1996; Soetens *et al.*, 2001; Dupré & Haguenauer-Tsapis, 2001; Krsmanović *et al.*, 2005; Shih *et al.*, 2002). The other is biosynthetic cargo, i.e., vacuolar proteins transported directly from the late Golgi to the anterograde and biosynthetic pathway, such as carboxypeptidase S (CPS), Phm5p, and Sna3p (Odorizzi *et al.*, 1998; Reggiori & Pelham, 2001). In mammalian cells, MVBs are also involved in budding of viruses and in lipid partitioning (Piper & Luzio, 2001).

ESCRT complexes function sequentially in the sorting of transmembrane proteins into the MVB pathway and in the formation of MVB vesicles (reviewed in Babst, 2005; Bowers & Stevens, 2005; Winter & Hauser, 2006). ESCRT-I localization to the MVB membrane depends on its interaction with the Vps27/Hse1 complex, also called ESCRT-0 (Bowers & Stevens, 2005). The Vps27/Hse1 complex is the sorting receptor for ubiquitinated cargo proteins at the MVB. The ESCRT-I complex binds to ubiquitinated cargo and activates ESCRT-II, although this activation mechanism is not yet understood. ESCRT-II in turn initiates the oligomerization of small coiled-coil proteins, resulting in the formation of the ESCRT-III complex, which concentrates MVB cargo (Babst *et al.*, 2002). ESCRT-III recruits the deubiquitinating enzyme Doa4p, which removes ubiquitin (Ub) from the cargo protein prior to sorting into the MVB vesicles (Amerik *et al.*, 2000). After protein sorting is completed, the AAA-type ATPase, Vps4p, binds to ESCRT-III and disassembles ESCRT-III in an ATP-dependent manner (Babst *et al.*, 1997, 1998).

The mammalian Hrs/STAM complex, equivalent to the Vps27/Hse1 complex in budding yeast, binds two deubiquitinating enzymes, the JAMM/MPN+ family member AMSH (associated molecule with the SH3 domain of STAM) and the ubiquitin**-**specific protease family member UBPY (ubiquitin isopeptidase Y) (McCullough *et al.*, 2004; Tanaka *et al.*, 1999; Row *et al.*, 2006). These two deubiquitinating enzymes may share redundant functions: the promotion of MVB cargo recycling by opposing ubiquitin ligase activity, and the deubiquitination of MVB cargo prior to lysosomal degradation. Earlier work demonstrated that AMSH counteracts the E3-ligase through deubiquitination of MVB cargo (McCullough *et al.*, 2004). Recent studies showed that AMSH binds to the ESCRT-III complex in a STAM-independent manner (Agromayor & Martin-Serrano, 2006; Tsang *et al.*, 2006), in support of the suggestion that AMSH might be a counterpart of Doa4p (Agromayor & Martin-Serrano, 2006). The function of UBPY is uncertain. One report concludes that UBPY negatively regulates degradation of the epidermal growth factor receptor (EGFR) (Mizuno *et al.*, 2005), which is downregulated via MVB sorting and lysosomal degradation. Others conclude that UBPY-mediated deubiquitination is essential for EGFR degradation (Row *et al.*, 2006; Alwan & van Leeuwen, 2007). UBPY also catalyses deubiquitination of Eps15, suggesting that it controls the level of ubiquitinated protein required for maintaining the morphology of the endosome (Mizuno *et al.*, 2006).

Recent completion of the *Sch. pombe* genome sequence revealed that class E Vps proteins were largely conserved in this species (Takegawa *et al.*, 2003). Nonetheless, the MVB pathway in *Sch. pombe* is still poorly understood. Our previous study identified six genes as suppressors of *ste12* (*sst* genes), including casein kinase II and a calcium transporter (Onishi *et al.*, 2003). Ste12p is a phosphatidylinositol 3-phosphate (PtdIns-3-P) 5-kinase, equivalent to *Sac. cerevisiae* Fab1p (McEwen *et al.*, 1999). Two class E *vps* genes are included among the *sst* genes. *sst4/vps27^+^* is a *VPS27* homologue and *sst6^+^* is similar to *VPS23* of *Sac. cerevisiae*. In addition, *sst2^+^* is a homologue of mammalian AMSH.

Here, we describe analyses of common phenotypes in class E mutants in fission yeast. Loss of these proteins resulted in mild defects in maturation of carboxypeptidase Y (CPY) and sorting into MVBs. This is the first report showing that the MVB pathway functions in *Sch. pombe* and that the roles of class E Vps proteins in MVB sorting are conserved.

## METHODS

### Strains, media and genetic methods.

*Sch. pombe* strains used in this study are listed in Table 1. Standard rich medium (YES) and synthetic minimal medium (MM) for growing *Sch. pombe* were used as described previously (Moreno *et al.*, 1991). *Sch. pombe* cells were transformed by the lithium acetate method or by electroporation as described previously (Okazaki *et al.*, 1990; Suga *et al.*, 2000; Suga & Hatakeyama, 2001; Morita & Takegawa, 2004). Standard genetic methods have been described previously (Alfa *et al.*, 1993).

### Pulse–chase and immunoblot analyses of the *Sch. pombe* Cpy1 protein.

Pulse–chase analysis and immunoprecipitation of the vacuolar CPY from *Sch. pombe* (Cpy1p) were carried out as previously described (Tabuchi *et al.*, 1997). Antibody incubations were performed using rabbit polyclonal antibody against *Sch. pombe* Cpy1p as described (Tabuchi *et al.*, 1997).

Colony blot assays to detect mislocalized Cpy1p were performed as previously described (Cheng *et al.*, 2002). Briefly, cells were spotted on nitrocellulose membranes and grown for 2 days at 30 °C. After removing cells by washing, the nitrocellulose membranes were subjected to immunodetection of Cpy1p using rabbit polyclonal antibodies raised against *Sch. pombe* Cpy1p (1 : 500 dilution), horseradish peroxidase-conjugated anti-rabbit IgG antiserum (Amersham Biosciences) and the Amersham ECL system.

### Gene disruptions.

The *sst4/vps27*^+^ locus (SPAC19A8.05C) was disrupted in the wild-type *Sch. pombe* strain by replacing an internal *sst4/vps27*^+^ gene fragment with the *Sch. pombe ura4*^+^ gene. To amplify the DNA fragment carrying the *sst4/vps27*^+^ gene from the complementing DNA, the following oligonucleotides were used: sense, 5′-ATACCGAGATGTGCTAAGCTGCCCGC-3′ and antisense, 5′-CAGACATGCATTGTCGATAA-3′. A 2.1 kbp fragment was recovered and ligated into pGEM-T Easy vector (Promega). An *Xba*I site within the cloned *sst4/vps27*^+^ open reading frame was digested and a 1.6 kbp *ura4^+^* gene was inserted.

To disrupt the *sst6*^+^ locus (SPAC11H11.01), the following oligonucleotides were used: sense, 5′-GAAAATGAAGCTCCTCCTGTTATCCCTGC-3′ and antisense, 5′-TCAAACAGCACTTCATACTTAATGTTCTGC-3′. A 1.4 kbp fragment was recovered and ligated into the pGEM-T Easy vector (Promega). Two *Hin*cII sites within the cloned *sst6*^+^ open reading frame were digested and the *ura4^+^* gene was inserted. A linearized DNA fragment carrying the disrupted *sst4/vps27*^+^ and *sst6*^+^ genes was used to transform wild-type haploid WA8 strains, and *ura*^+^ transformants were selected. To confirm that one of the *sst4/vps27*^+^ and *sst6*^+^ genes had been disrupted, *ura*^+^ transformants were analysed by Southern blot and PCR to verify correct integration of the deletion constructs.

The *sst2^+^* deletion was generated as follows: 0.6 kbp fragments carrying the promoter and terminator were amplified by PCR and then cloned sequentially into *Xho*I and *Hin*dIII sites (promoter) and *Eco*RI and *Bam*HI sites (terminator) of pBS loxP-ura4-loxP (Iwaki & Takegawa, 2004), followed by amplification of the disruption cassette and transformation of yeast. *ura*^+^ transformants were analysed by PCR and *ura4^+^* was removed by Cre-mediated recombination using pREP41-Cre (Iwaki & Takegawa, 2004).

### Plasmid constructions.

pREP41-Ub-GFP-SpCPS was constructed as follows. The 225 bp fragment encoding a single ubiquitin molecule from the *ubi4* gene was amplified and cloned into the *Nde*I and *Sal*I sites of pREP41, and confirmed by sequence analysis. A *CPS1* homologue, SPAC24C9.08 (SpCPS), was amplified by PCR and subcloned into pTN54, a derivative of the thiamine-repressible expression vector pREP41 (Nakamura *et al.*, 2001), resulting in plasmid pTN54/SpCPS, which expresses an N-terminal GFP-tagged *Sp*CPS. The GFP–*Sp*CPS fusion was amplified by PCR and cloned into the *Sal*I and *Bam*HI sites of pREP41 containing the ubiquitin sequence, resulting in plasmid pREP41-Ub-GFP-SpCPS. To generate a *ura4^+^* marker plasmid, pREP41-Ub-GFP-SpCPS was digested with *Pst*I and *Bam*HI. A fragment containing the *nmt1* promoter and the Ub–GFP–SpCPS ORF was recovered, and then cloned into the corresponding sites of pREP42.

pAU/nmt41-RFP-Ptn1 was constructed as follows: codon usage of RFP (red fluorescent protein) was optimized for *Sch. pombe* to generate pRFPm1-2 by GENEART. The attenuated *nmt1* promoter from pREP41, the RFP ORF from pRFPm1-2 and the *ptn1* ORF from genomic DNA were amplified by PCR, digested with *Xho*I and *Eco*RI (*nmt1* promoter), *Eco*RI and *Bam*HI (RFP) and *Bam*HI and *Not*I (*ptn1*), respectively, and then sequentially cloned into the corresponding sites of pAU-SK.

pAL(map3-GFP), a multicopy plasmid for expression of Map3–GFP, was obtained from Dr C. Shimoda (Morishita *et al.*, 2002).

### Vacuole staining.

Vacuolar membranes were labelled with FM4-64 (Vida & Emr, 1995). Cells were grown to exponential phase in YES medium at 30 °C and 500 μl cells was then incubated in medium containing 8 μM FM4-64 for 30 min at 30 °C. The cells were then centrifuged at 13 000 ***g*** for 1 min, washed by resuspending in YES to remove free FM4-64 and collected by centrifugation at 13 000 ***g*** for 1 min. Cells were then resuspended in YES and incubated for 90 min at 30 °C before microscopic observation. Stained cells were observed using a fluorescence microscope (model BX-60; Olympus).

### Internalization assay using FM4-64.

Exponentially growing cells in YES medium were incubated with 16 μM FM4-64 on ice for 30 min to label the plasma membrane. Cells were then washed with ice-cold fresh medium to remove excess dye, and resuspended in ice-cold fresh medium. Cells were then incubated at 30 °C and a small aliquot was withdrawn after adequate incubation for microscopic observation.

### Analysis of fluid-phase endocytosis.

Fluid-phase endocytosis was observed microscopically after cells were treated with Lucifer Yellow CH (Sigma-Aldrich). Staining with Lucifer Yellow CH was performed as described previously (Murray & Johnson, 2001). Briefly, 1 ml of exponentially growing cells in YES medium was collected by centrifugation, washed twice with fresh medium and resuspended in 0.5 ml YES medium containing 5 mg ml^−1^ Lucifer Yellow CH. Cells were incubated at 30 °C for 60 min with shaking and then washed three times with fresh medium. Labelled cells were then subjected to microscopic observation.

### Fluorescence microscopy.

Cells were observed with an Olympus BX-60 fluorescence microscope using a U-MGFPHQ filter set (for GFP), U-MWBV filter set (for Lucifer Yellow CH) or U-MIG filter set (for RFP and FM 4-64; all filters Olympus). Images were captured with a SenSys Cooled CCD camera using MetaMorph (Roper Scientific), and were saved as Adobe Photoshop files on a Macintosh G4 computer.

## RESULTS

### The *sst4/vps27*^+^ and *sst6*^+^ genes encode homologues of *Sac. cerevisiae* class E Vps proteins

**Fig. 1. f1:**
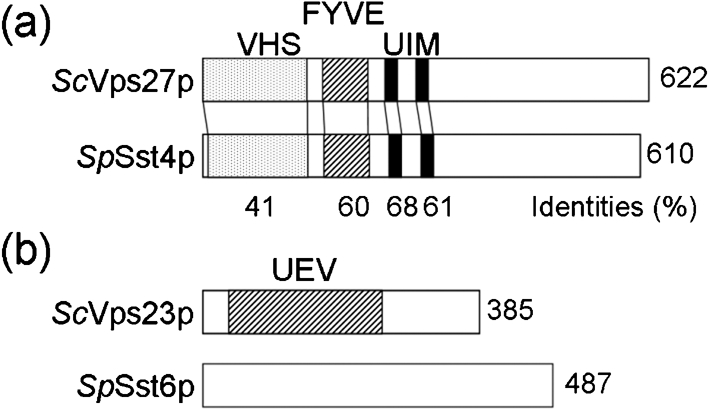
Characterization of *sst4/vps27*^+^ and *sst6*^+^ genes. (a) Domain structures of the *Sch. pombe* Sst4/Vps27 (*Sp*Sst4p) and *Sac. cerevisiae* Vps27 (*Sc*Vps27p) proteins. The shaded, hatched and closed boxes indicate the VHS, FYVE and UIM domains, respectively. (b) Domain structures of the *Sch. pombe* Sst6 (*Sp*Sst6p) and *Sac. cerevisiae* Vps23 (*Sc*Vps23p) proteins. *Sc*Vps23p possesses a UEV domain (hatched), similar to the catalytic domain of the E2 UBC enzyme, but *Sp*Sst6p has no domains defined by the SMART or Pfam databases.

In a previous study, *sst4* and *sst6* were identified as *vps27*/SPAC19A8.05c and SPAC11H11.01, respectively (Onishi *et al.*, 2003). The *sst4/vps27*^+^ gene was found to be homologous to the *Sac. cerevisiae VPS* gene, *VPS27*, and *sst6^+^* was similar to *VPS23*. Overall, *Sac. cerevisiae* Vps27p and *Sch. pombe* Sst4/Vps27p are similar in size (622 and 610 aa, respectively) and share approximately 50 % amino acid sequence similarity (Fig. 1a[Fig f1]). In addition, both Vps27p and Sst4/Vps27p have similar predicted domain structures, having VHS (Vps27p, Hrs, and STAM), FYVE and UIM (ubiquitin-interacting motif) domains. The VHS domain was originally identified in a database screen of sequences in signal transduction proteins (Lohi *et al.*, 2002). The VHS domain of Vps27p appears to play a role in Hse1p-associated endocytosis of a selected set of receptor molecules (Shih *et al.*, 2002; Bilodeau *et al.*, 2002). The FYVE domain of *Sac. cerevisiae* Vps27p has been shown to bind to liposomes that contain PtdIns-3-P, but not to liposomes that contain other phosphoinositides, indicating that the FYVE domain of Vps27p specifically binds to PtdIns-3-P *in vitro* (Burd & Emr, 1998). The UIM domain was determined based on sequences identified in the subunit of the 26S proteasome that interacts with polyubiquitin (Young *et al.*, 1998).

Although the amino acid sequence of Sst6 revealed a relatively low overall level of similarity with *Sac. cerevisiae* Vps23p, the 60 C-terminal amino acids are conserved with mammalian TSG101 (Babst *et al.*, 2000; Bishop & Woodman, 2001). The ubiquitin E2 variant (UEV) domains reside in the N-terminal regions of Vps23p and TSG101, and share homology with the catalytic domain of ubiquitin-conjugating enzymes (UBC). However, Vps23p and TSG101 are unlikely to catalyse ubiquitination because both proteins have substitutions for the cysteine residue that forms a reversible covalent bond with ubiquitin in E2 enzymes (Babst *et al.*, 2000). Interestingly, the Sst6 protein does not contain a UEV domain (Fig. 1b[Fig f1]).

### Disruption of *sst4/vps27*^+^ and *sst6*^+^ results in moderate vacuolar protein sorting defects

**Fig. 2. f2:**
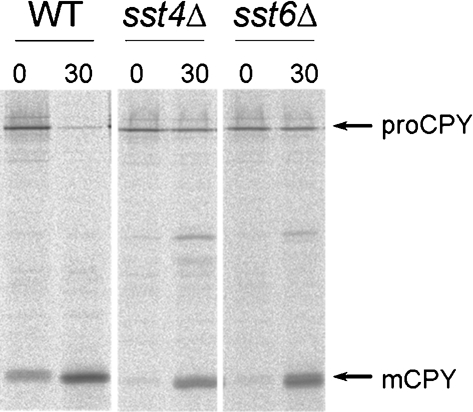
Processing of *Sp*CPY in *sst4/vps27*Δ and *sst6*Δ cells. Wild-type (WT), *sst4/vps27*Δ (*sst4*Δ) and *sst6*Δ cells were pulse-labelled with Express-^35^S (NEN) for 15 min at 30 °C, and chased for 30 min. The immunoprecipitates were separated on an SDS polyacrylamide gel (10 %). Autoradiograms of the fixed dried gels are shown. The positions of proCPY (110 kDa) and mature CPY (mCPY, 32 kDa) are indicated.

To determine whether the *sst4/vps27*^+^ and *sst6*^+^ genes are required for vacuolar protein trafficking in *Sch. pombe*, *sst4/vps27* and *sst6* null mutants were constructed in a wild-type (WA8) background. We have previously reported the isolation and characterization of a vacuolar marker protein, a carboxypeptidase from *Sch. pombe* (Cpy1p) (Tabuchi *et al.*, 1997). To determine whether *Sch. pombe* Sst4/Vps27p and Sst6p are required for vacuolar protein transport, the sorting of Cpy1p in the *sst4/vps27*Δ and *sst6*Δ mutants was analysed by pulse–chase experiments. During synthesis, Cpy1p undergoes a characteristic modification, a change in its apparent molecular mass. After the 15 min pulse period, the endoplasmic reticulum- and Golgi-specific precursor form (proCPY) and a small amount of the vacuole-specific mature form (mCPY) were labelled in wild-type cells, and after the 30 min chase, all Cpy1p was transported to the vacuole and matured, indicating that Cpy1p was properly delivered to the vacuole (Fig. 2). The *sst4/vps27*Δ and *sst6*Δ null mutants showed a sorting defect for Cpy1p. After the 30 min chase, approximately 30 % of the proCPY was still detected, while most of the remaining Cpy1p was processed to the mature form in *sst4/vps27*Δ and *sst6*Δ cells, indicating that it had been transported to a compartment containing active vacuolar hydrolases (Fig. 2). Colony blot assay indicated that a fraction of CPY was secreted into the medium in these mutants (Fig. 7a[Fig f7]). These results indicate that the Sst4/Vps27 and Sst6 proteins are required for efficient delivery of Cpy1p to the vacuole in *Sch. pombe*.

### Vacuolar morphology and endocytosis in *sst4/vps27*Δ and *sst6*Δ

**Fig. 3. f3:**
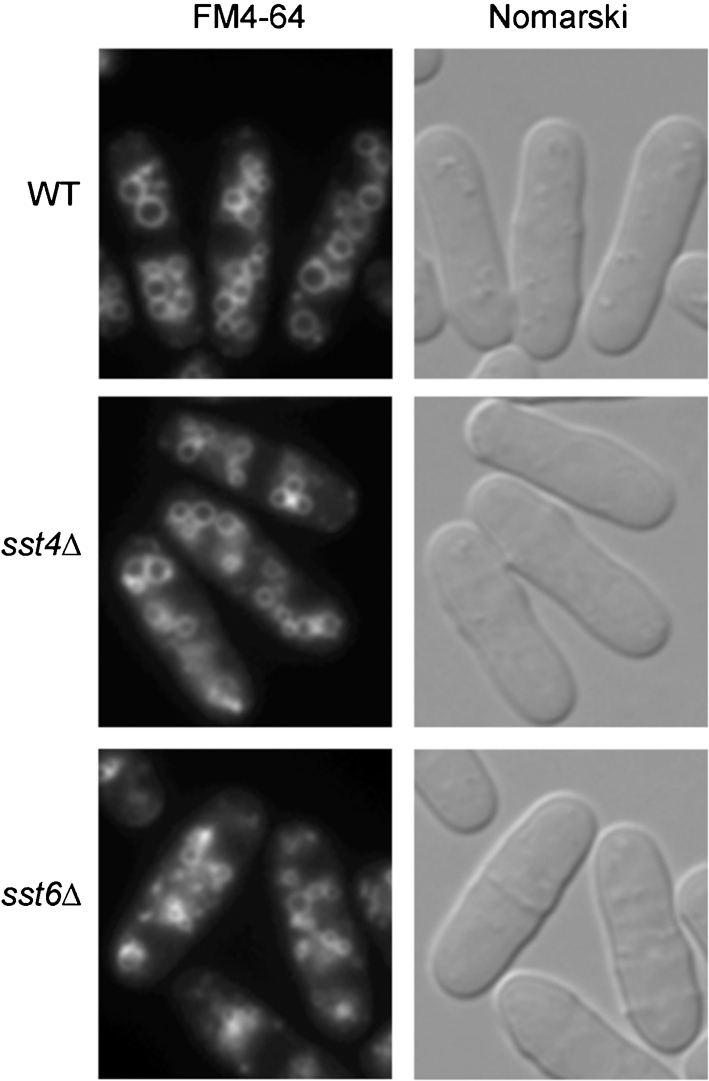
Vacuolar morphology in *sst4/vps27*Δ and *sst6*Δ mutants. Vacuoles of wild-type (WT), *sst4/vps27*Δ (*sst4*Δ) and *sst6*Δ cells were stained with FM4-64.

*Sac. cerevisiae* class E *vps* mutants accumulate FM4-64 in the vacuolar membrane and class E compartment adjacent to the vacuole (Raymond *et al.*, 1992; Vida & Emr, 1995), and are defective in endocytosis (Piper *et al.*, 1995). Staining with FM4-64 revealed that vacuoles in *sst4/vps27*Δ and *sst6*Δ were mostly similar in size to wild-type, but that some of them were smaller (Fig. 3). They were nearly normal compared to those found in *ypt7*Δ, which has fragmented vacuoles because of the lack of vacuolar fusion (Iwaki *et al.*, 2004). Structures corresponding to class E compartment were indistinguishable, because of the size and number of vacuoles. However, transmission electron microscopy revealed that class E *vps* mutants accumulated aberrant membranous structures (Supplementary Fig. S1, available with the online version of this paper).

**Fig. 4. f4:**
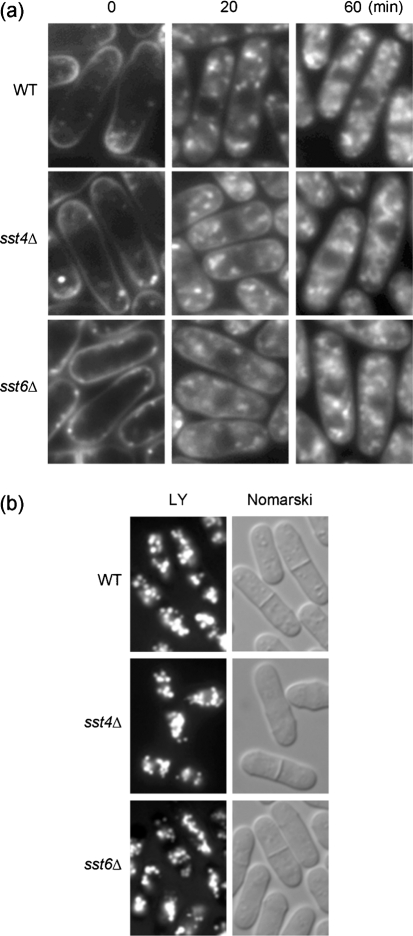
Analyses of endocytosis. (a) Time-course of FM4-64 internalization via the endocytic pathway. Living wild-type (WT), *sst4/vps27*Δ (*sst4*Δ) or *sst6*Δ cells were labelled with 16 μM FM4-64 on ice for 30 min and then incubated at 30 °C for the indicated periods. (b) Accumulation of Lucifer Yellow CH. Wild-type (WT), *sst4/vps27*Δ (*sst4*Δ) or *sst6*Δ cells were incubated for 60 min at 30 °C in YES medium containing 5 mg ml^−1^ Lucifer Yellow CH (LY). Cells were then washed and observed by fluorescence microscopy.

To examine the effect of the loss of Sst4/Vps27p and Sst6p on the endocytic pathway, *sst4/vps27*Δ and *sst6*Δ cells were labelled with FM4-64 on ice, followed by incubation at 30 °C. This fluorescent dye initially stains the plasma membrane and is then internalized and delivered to the vacuolar membrane in a time-, energy- and temperature-dependent manner (Vida & Emr, 1995). After labelling of wild-type, *sst4/vps27*Δ and *sst6*Δ cells with FM4-64 for 30 min on ice, the dye stained the plasma membrane and septa, and several punctate patches were observed near the plasma membrane (Fig. 4a[Fig f4]). Following incubation at 30 °C, the FM4-64 dye was gradually transported out of the peripheral membranes, and the patches were then observed to be distributed in the cytoplasm. After incubation at 30 °C for 20 min, FM4-64 predominantly labelled the prevacuolar compartments. Staining of the plasma membrane was not detected in wild-type cells. In *sst4/vps27*Δ and *sst6*Δ cells, we observed pronounced staining of the plasma membranes and prevacuolar compartments (Fig. 4a[Fig f4]). After a 60 min chase at 30 °C, FM4-64 was transported in part to the vacuolar membrane, such that the staining patterns of the *sstΔ* mutants could not be distinguished from those observed in wild-type. These results demonstrate that the FM4-64 dye was slowly internalized in *sst4/vps27*Δ and *sst6*Δ cells.

To determine whether *sstΔ* cells are also defective fluid-phase endocytosis, accumulation of Lucifer Yellow CH was observed. Wild-type, *sst4/vps27*Δ and *sst6*Δ cells were incubated in Lucifer Yellow CH for 1 h at 30 °C. Vacuolar accumulation of Lucifer Yellow CH was observed in wild-type as expected, but also in the *sst4/vps27*Δ and *sst6*Δ mutants (Fig. 4b[Fig f4]). These results indicate that Sst proteins are not essential for fluid-phase endocytosis in *Sch. pombe*. Vacuoles containing Lucifer Yellow CH in *sst*Δ cells were similar to those in wild-type, supporting the view that these mutants have nearly normal vacuoles.

**Fig. 5. f5:**
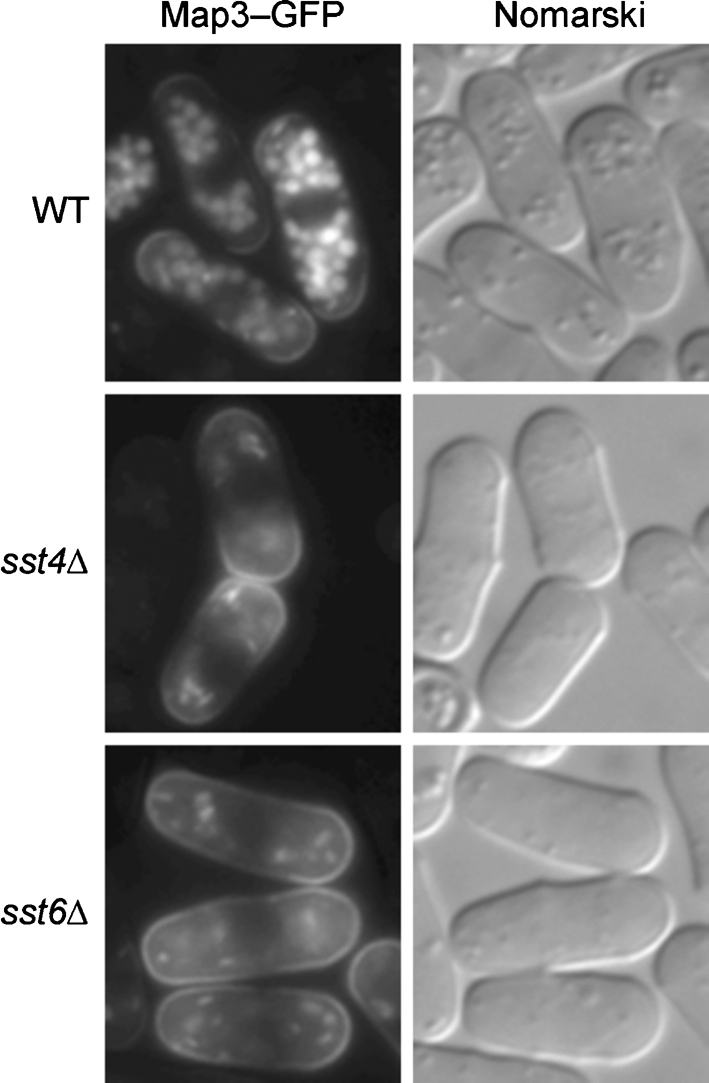
Localization of Map3–GFP in homothallic cells. Cells containing pAL(map3-GFP) were grown in 5 ml MM medium without Leu for 20 h, and then observed under a fluorescence microscope.

Map3p is the mating pheromone M-factor receptor expressed in *h^+^* strains. The internalization of Map3–GFP occurs in homothallic cells that do not undergo successful conjugation when subjected to nitrogen starvation (Hirota *et al.*, 2001). Map3–GFP was expressed under the control of the *map3* promoter on a multicopy plasmid, resulting in overexpression. Wild-type homothallic cells overexpressing Map3–GFP formed aggregates even when not starved for nitrogen (data not shown), probably because of the increased mating efficiency. Map3–GFP was found in the vacuoles and partly on the cell surface of unconjugated wild-type cells (Fig. 5), suggesting that Map3–GFP was internalized and degraded. Map3–GFP-overexpressing *sst4/vps27*Δ or *sst6*Δ cells did not form aggregates, and Map3–GFP localized mostly on the cell surface (Fig. 5). This observation suggests that internalization of plasma membrane protein**s** might be inhibited in these mutants.

### Sst4/Vps27p and Sst6p are required for MVB sorting

CPS is one of the most studied MVB cargo proteins, and the Ub–GFP–CPS fusion protein has been used as a marker for MVB sorting in *Sac. cerevisiae* (Katzmann *et al.*, 2004). To test the important question of whether the MVB is found in *Sch. pombe* and whether Sst4/Vps27p and Sst6p play significant roles in MVB sorting, we searched the *Sch. pombe* genome database for a CPS homologue. One CPS homologue, SPAC24C9.08 (*Sp*CPS) was found and, therefore, GFP–*Sp*CPS was constructed and expressed, because GFP–CPS is also known as a good indicator of the MVB in *Sac. cerevisiae* (Odorizzi *et al.*, 1998). Ubiquitin chains are added to lysine residues, and K_8_ of the N-terminal cytoplasmic domain is known to be ubiquitinated in *Sac. cerevisiae* CPS (Katzmann *et al.*, 2001). *Sp*CPS has four lysine residues in the putative N-terminal cytoplasmic domain, K_16_, K_19_, K_32_ and K_33_. Ubiquitin chains may be added to one or more of these residues. However, GFP–*Sp*CPS was not effectively sorted into vacuoles even in wild-type. It is possible that GFP may inhibit ubiquitination of *Sp*CPS or interfere with normal interaction with Sst4/Vps27p.

In an attempt to identify an MVB marker protein, SPBC713.07c was found to be a homologue of *Sac. cerevisiae* Phm5p (Supplementary Fig. S2a, available with the online version of this paper), which is also transported into vacuoles via the MVB pathway (Reggiori & Pelham, 2001). A GFP–SPBC713.07c fusion protein was expressed in wild-type cells, but the fusion protein was not localized in the vacuoles (Supplementary Fig. S2b).

**Fig. 6. f6:**
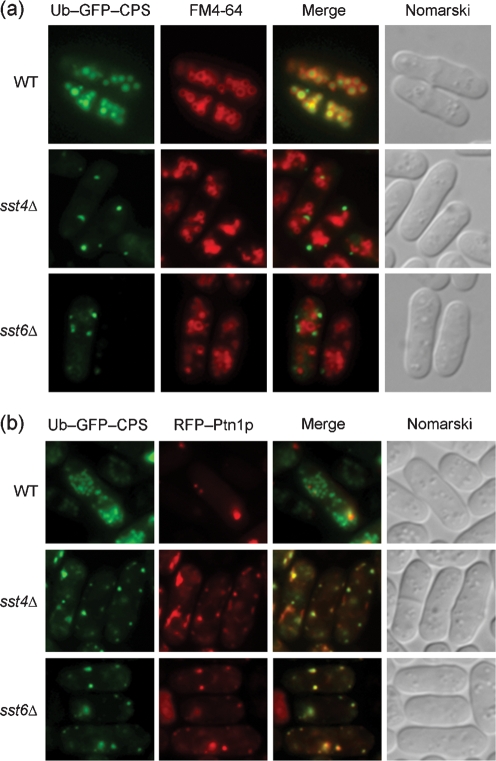
Localization of Ub–GFP–*Sp*CPS in *sst4/vps27*Δ and *sst6*Δ cells. (a) Sorting of Ub–GFP–*Sp*CPS into vacuoles is inhibited in *sst4/vps27*Δ and *sst6*Δ. Cells containing pREP41-Ub-GFP-*Sp*CPS were grown in MM medium without Leu and thiamine for 20 h, after which vacuoles were labelled with FM4-64. Wild-type (WT), *sst4/vps27*Δ (*sst4*Δ) and *sst6*Δ cells are shown. (b) Ub–GFP–*Sp*CPS is trapped in endosomes in *sst4/vps27*Δ and *sst6*Δ cells. Cells expressing Ub–GFP–*Sp*CPS and RFP–Ptn1p were grown in MM medium without Leu, Ura and thiamine for 20 h. Wild-type (WT), *sst4/vps27*Δ (*sst4*Δ) and *sst6*Δ cells are shown.

Therefore, a single ubiquitin molecule was fused to the N-terminus of GFP–*Sp*CPS, and Ub–GFP–*Sp*CPS was expressed in *sst4/vps27*Δ and *sst6*Δ mutants (Fig. 6a[Fig f6]). Wild-type cells expressing Ub–GFP–*Sp*CPS exhibited a vacuolar pattern of fluorescence corresponding to the staining pattern of FM4-64, indicating that it was sorted into MVB vesicles and transported to the vacuoles. Ub–GFP–*Sp*CPS exhibited punctate fluorescence and separated from vacuoles in *sst4/vps27*Δ and *sst6*Δ mutants (Fig. 6a[Fig f6]).

PtdIns 3,4,5-triphosphate 3-phosphatase, Ptn1p, is reported to accumulate on the endosome (Mitra *et al.*, 2004). To clarify localization of Ub–GFP–*Sp*CPS, RFP (red fluorescent protein) was fused to the N-terminus of Ptn1p and co-expressed under an attenuated *nmt1* promoter. In wild-type cells, these two proteins showed distinct patterns of fluorescence (Fig. 6b[Fig f6]). Most of the fluorescent dots of Ub–GFP–*Sp*CPS merged with RFP–Ptn1p in *sst4/vps27*Δ and *sst6*Δ mutants (Fig. 6b[Fig f6]), suggesting that it localizes primarily to endosomes, and that sorting into MVB is inhibited.

### Mutations in putative class E *vps* genes also cause MVB sorting defects

**Fig. 7. f7:**
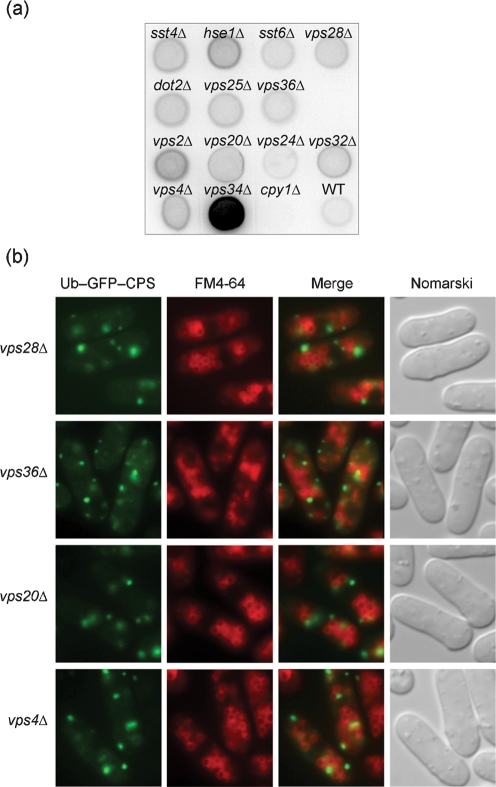
Phenotypic analyses of various class E mutants. (a) Secretion of CPY was determined using a colony blot assay. The membranes were subjected to immunoblotting with rabbit anti-CPY. Wild-type (WT), *vps34*Δ (positive control) and MTD2 (*cpy1*Δ, negative control) are included for comparison. (b) Localization of Ub–GFP–*Sp*CPS was compared with vacuolar staining of FM4-64. Cells were processed as described in the legend to Fig. 6(a). *vps28*Δ, *vps36*Δ and *vps20*Δ are representative of ESCRT-I, ESCRT-II and ESCRT-III, respectively. All other mutants exhibited the same patterns of fluorescence.

Since *VPS23* and *VPS27* are classified as class E *vps* genes in *Sac. cerevisiae* (Raymond *et al.*, 1992), the *Sch. pombe* homologues were deleted and the resultant mutants tested for defects in CPY maturation and MVB sorting. Disruptants of putative class E *vps* genes, consisting of ESCRT machinery, were constructed (Table 2). While *VPS37* homologues were not found in the fission yeast genome, a *VPS22* homologue, *dot2^+^*, has been reported to be a homologue of the human transcription factor EAP30, which negatively regulates meiotic spindle pole body maturation (Jin *et al.*, 2005). Disruption of all of these *vps* genes resulted in mild defects in CPY maturation (data not shown), and missorting of a fraction of CPY into the medium (Fig. 7a[Fig f7]), as observed for the *sst4/vps27*Δ and *sst6*Δ mutants. A disruption mutant of *cpy1* encoding CPY, *cpy1*Δ, was used as a negative control (Tabuchi *et al.*, 1997). *vps34*Δ was used as positive control secreting strong levels of CPY (Cheng *et al.*, 2002). Localization analysis of Ub–GFP–*Sp*CPS was also undertaken for these mutants (Fig. 7b[Fig f7]). None was found able to sort Ub–GFP–*Sp*CPS into vacuoles, suggesting that these genes are required for sorting into the MVB.

### *sst2^+^* is a class E *vps* gene in fission yeast

While recent studies found that a deubiquitinating enzyme, AMSH, is also essential for the MVB pathway in mammals (McCullough *et al.*, 2004; Agromayor & Martin-Serrano, 2006), it was not found in *Sac. cerevisiae,* which has a different deubiquitinating enzyme, Doa4p (Amerik *et al.*, 2000). *Sch. pombe* has an AMSH homologue, previously identified as *sst2^+^*(Onishi *et al.*, 2003). Apparent PXXP motifs, required for interaction with STAM (Tanaka *et al.*, 1999), were not found in Sst2p, although these proteins share 31 % identity.

**Fig. 8. f8:**
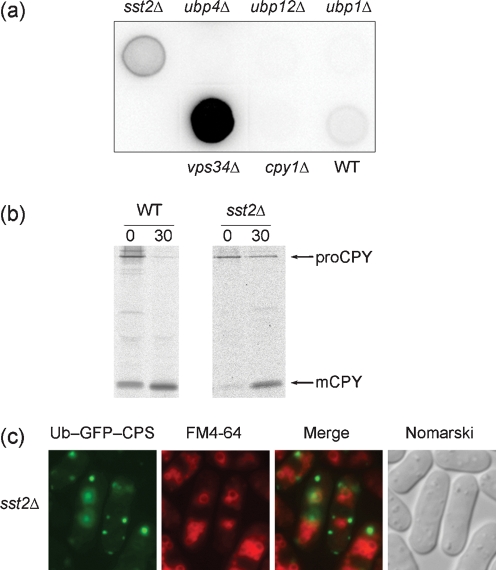
Characterization of *sst2*^+^. (a) Secretion of CPY was determined using a colony blot assay as described in the legend to Fig. 4(a). Wild-type (WT), *vps34*Δ and MTD2 (*cpy1*Δ) are included for comparison. (b) Processing of *Sp*CPY in the *sst2*Δ mutant. *sst2*Δ cells were processed as described in the legend to Fig. 2. The positions of proCPY (110 kDa) and mature CPY (mCPY, 32 kDa) are indicated. (c) Localization of Ub–GFP–*Sp*CPS in the *sst2*Δ mutant. Cells were processed as described in the legend to Fig. 6(a).

The *Sac. cerevisiae doa4* mutant was found capable of normal CPY delivery to vacuoles (Amerik *et al.*, 2000), but exhibited aberrant sorting into the MVB (Reggiori & Pelham, 2001), indicating a contribution to the MVB pathway. While ubiquitin C-terminal hydrolases in *Sch. pombe* share sequence similarity with one another, three proteins exhibited relatively high homology with Doa4p: Ubp1p/SPCC16A11.12c, Ubp4p/SPBC18H10.08c and Ubp12p/SPCC1494.05 (Table 2). Ubp4p has been annotated as a Doa4p homologue in the genome database. Disruption mutants of these four genes encoding putative deubiquitinating enzymes were constructed. While the *sst2*Δ mutant was able to secrete CPY (Fig. 8a[Fig f8]), it exhibited defects in CPY maturation (Fig. 8b[Fig f8]) and impaired sorting into the MVB (Fig. 8c[Fig f8]). CPY secretion and mislocalization of Ub–GFP–*Sp*CPS were not detected in the *ubp1*Δ, *ubp4*Δ, or *ubp12*Δ mutants (Fig. 8a[Fig f8]), indicating that disruption of these *ubp* genes did not result in a class E phenotype. However, a contribution of Ubp proteins to MVB sorting cannot be ruled out, because a ubiquitin-fused marker protein is normally transported into vacuoles in a *Sac*. *cerevisiae doa4*Δ mutant (Reggiori & Pelham, 2001).

## DISCUSSION

In the present study, we found that sorting defects in the *sst4/vps27*Δ and *sst6*Δ *Sch. pombe* mutants are relatively modest; 30 % or less of CPY was not processed to the mature form (Fig. 2). The modest sorting defect, together with the delay of transport of FM4-64 to the vacuolar membranes (Fig. 4), suggests that *sst4/vps27*Δ and *sst6*Δ mutants are not completely defective in vacuolar transport, but rather exhibit a kinetic defect in transport out of the prevacuolar endosome-like compartments. Functional conservation of these proteins was expected from homology to budding yeast homologues. In agreement with this idea, a biosynthetic MVB cargo, Ub–GFP–*Sp*CPS, was trapped in the endosomes in these mutants (Fig. 6). Transmission electron microscopy revealed that class E *vps* mutants accumulated aberrant membranous structures (Supplementary Fig. S1, available with the online version of this article), although we have not confirmed that these compartments correspond to *Sac. cerevisiae* class E compartments.

The *Sch. pombe* Sst4/Vps27 protein contains a UIM domain, similar to *Sac. cerevisiae* Vps27p (Fig. 1a[Fig f1]). It was recently shown that the UIM domain of *Sac. cerevisiae* Vps27p binds to mono-ubiquitin through Ile44 of ubiquitin (Shih *et al.*, 2002). Cell-surface transmembrane proteins such as G-protein-coupled receptors and transporters in *Sac. cerevisiae* are modified with ubiquitin in response to ligand binding. Ubiquitination serves to trigger rapid internalization and degradation of these proteins in the vacuole (Hicke, 1999). Several transmembrane proteins, including CPS and Phm5p, which are delivered from the Golgi to the endosome, are also ubiquitinated in *Sac. cerevisiae* (Reggiori & Pelham, 2001). The UIM domain of Sst4/Vps27p seems to be required for efficient sorting of cargo from both the biosynthetic and the endocytic pathways destined for delivery to the vacuole in *Sch. pombe*. In contrast to the Sst4/Vps27 protein, the *Sch. pombe* Sst6 protein lacks the UEV domain, which in *Sac. cerevisiae* Vps23p and in mammalian TSG101 has been shown to bind ubiquitin (Babst *et al.*, 2000; Katzmann *et al.*, 2001). The UEV domain of Vps23p is required for ESCRT-I to bind ubiquitin *in vitro* (Katzmann *et al.*, 2001). Our results show that *Sch. pombe* Sst6p is required for the vacuolar protein sorting pathway and for MVB sorting. Therefore, the UEV domain is not essential for Sst6p function. Further analysis will be required to determine whether Sst6p forms a larger protein complex and whether Sst6p can bind to ubiquitin in the absence of the UEV domain.

We also found that other class E Vps proteins are required for maturation of CPY and sorting into MVBs (Fig. 7), indicating that the roles of class E Vps proteins are conserved in fission yeast. However, there are differences between fission yeast and budding yeast. *Sch. pombe* has an AMSH homologue, Sst2p, which is not found in *Sac. cerevisiae*. Lack of Sst2p resulted in a phenotype similar to that exhibited by the *sst4/vps27*Δ mutant and by other class E mutants (Fig. 8), suggesting that Sst2p may be a component of the ESCRT complex. Sst2p might play a crucial role for endosomal function, such as regulation of MVB sorting through deubiquitination of ubiquitinated ESCRT proteins. Mammalian Hrs and STAM are ubiquitinated proteins (Katz *et al.*, 2002; Polo *et al.*, 2002), raising the possibility that Sst4/Vps27p and Hse1p might be ubiquitinated and then become substrates for Sst2p. blast searches using *Sch. pombe* Ubp proteins as the query sequence indicated that Ubp4p is the most plausible candidate of a Doa4p homologue in *Sch. pombe*, as annotated by the genome database. It remains to be elucidated whether Ubp4p contributes to the deubiquitination of MVB cargo and the regulation of endosomal functions through protein deubiquitination on the endosomes. In addition, homologues of Vps37p could not be found in the fission yeast genome by blast searches (Takegawa *et al.*, 2003). While recent experimental interaction studies have identified the human orthologues of *VPS37* (Bache *et al.*, 2004; Eastman *et al.*, 2005), the low levels of sequence identity were not sufficient to identify these homologues based on sequence alone. If *Sch. pombe* possesses a molecule with a function similar to that of Vps37p, its amino acid sequence will differ significantly from that of the *Sac. cerevisiae* and human Vps37ps.

As of this writing, there are few reports on the internalization and ubiquitination of cell-surface transmembrane proteins in *Sch. pombe*. One candidate molecule for an MVB cargo is the M-factor receptor Map3p. However, it has been already shown that internalization of Map3p is independent of ubiquitin (Hirota *et al.*, 2001). Our results indicate that *sst4/vps27*Δ and *sst6*Δ exhibit impaired internalization of Map3–GFP prior to MVB sorting (Fig. 4). Interestingly, depletion of ESCRT-II did not influence endocytosis of a mammalian EGFR (Bowers *et al.*, 2006). Our results show that sorting of biosynthetic MVB cargo is dependent on ESCRT-II in fission yeast, while it remains to be determined whether the endocytic MVB pathway depends on ESCRT-II or not. In order to address this question, it is essential to identify endocytic MVB marker proteins. We are currently examining the roles of ubiquitination with respect to the process of internalization of cell-surface transmembrane proteins in *Sch. pombe*. Future analyses are likely to provide important insights into the molecular details of MVB formation as well as ubiquitin-dependent protein trafficking in *Sch. pombe* cells.

## Figures and Tables

**Table 1. t1:** List of *Sc. pombe* strains used in this study

**Strain**	**Genotype**	**Reference or source**
WA8 (THP17)	*h*^90^*leu1 ura4-D18 ade6-M210*	Koga *et al.* (2004)
KJ100-7B	*h*^90^*leu1 ura4-D18*	Dr K. Tanaka (Tokyo University)
ARC039	*h*^−^*leu1-32 ura4-C190T*	Asahi Glass Co. Ltd
*vps34*Δ	*h^+^ leu1-32 ura4-D18 ade6-M216 vps34* : : *ura4^+^*	Takegawa *et al.* (1995)
MTD2	*h^+^ leu1-32 his2 ura4-D18 ade6-M216 cpy1* : : *ura4^+^*	Tabuchi *et al.* (1997)
*sst4*Δ	WA8 *vps27* : : *ura4^+^*	This study
*hse1*Δ	WA8 *hse1* : : *LEU2*	This study
*sst6*Δ	WA8 *sst6* : : *ura4^+^*	This study
*vps28*Δ	WA8 *vps28* : : *ura4^+^*	This study
*dot2*Δ	KJ100-7B *dot2* : : *ura4^+^*	This study
*vps25*Δ	KJ100-7B *vps25* : : *ura4^+^*	This study
*vps36*Δ	KJ100-7B *vps36* : : *ura4^+^*	This study
*vps2*Δ	ARC039 *vps2* : : *LEU2*	This study
*vps20*Δ	WA8 *vps20* : : *ura4^+^*	This study
*vps24*Δ	WA8 *vps24* : : *ura4^+^*	This study
*vps32*Δ	WA8 *vps32* : : *ura4^+^*	This study
*vps4*Δ	KJ100-7B *vps4* : : *ura4^+^*	This study
*sst2*Δ	KJ100-7B *sst2*Δ : : *loxP*	This study
*ubp1*Δ	ARC039 *ubp1* : : *ura4^+^*	This study
*ubp4*Δ	ARC039 *ubp4* : : *ura4^+^*	This study
*ubp12*Δ	ARC039 *ubp12* : : *ura4^+^*	This study

**Table 2. t2:** ESCRT complexes and deubiquitinating enzyme homologues in *Sch. pombe*

**Complex**	Homologue*	***Sch. pombe***	**E-value†**
Vps27/Hse1	*VPS27*	*sst4/vps27^+^*/SPAC19A8.05	7.8e–61
	*HSE1*	*hse1^+^*/SPBC1734.08	6.3e–59
ESCRT-I	*VPS23/STP22*	*sst6^+^*/SPAC11H11.01	2.5e–07
	*VPS28*	*vps28^+^*/SPAC1B3.07	1.3e–35
	*VPS37*	---	
ESCRT-II	*VPS22/SNF8*	*dot2^+^*/SPBC651.05c	2.3e–36
	*VPS25*	*vps25^+^*/SPBC4B4.06	6.3e–16
	*VPS36*	*vps36^+^*/SPBC3B9.09	2.5e–27
ESCRT-III	*VPS2/DID4*	*vps2^+^*/SPAC4F8.01	2.2e–49
	*VPS20*	*vps20^+^*/SPBC215.14c	1.3e–19
	*VPS24*	*vps24^+^*/SPAC9E9.14	1.1e–27
	*VPS32/SNF7*	*vps32^+^*/SPBC215.14	2.3e–38
Vps4	*VPS4*	*vps4^+^*/SPAC2G11.06	2.1e–145
Deubiquitinating enzyme	hAMSH	*sst2^+^*/SPAC19B12.10	2.7e–56
	*DOA4*	*ubp1^+^*/SPCC16A11.12c	3.9e–50
		*ubp4^+^*/SPBC18H10.08c	2.6e–49
		*ubp12^+^*/SPCC1494.05	2.7e–56

*Sac. cerevisiae homologues are indicated with the exception of human AMSH (hAMSH). †The fission yeast proteins were identified by screening the *Sch. pombe* genome database for new homologues of the known *Sac. cerevisiae* proteins using the blast program for sequence alignments (http://www.genedb.org/genedb/pombe/blast.jsp). Probability scores are shown as E-values. For Sst2p, the human AMSH protein sequence was used as a query.

## Abbreviations

- AMSH, associated molecule with the SH3 domain of STAM
- CPS, carboxypeptidase S
- CPY, carboxypeptidase Y
- EGFR, epidermal growth factor receptor
- ESCRT, endosomal sorting complex required for transport
- GFP, green fluorescent protein
- MVB, multivesicular body
- PtdIns, phosphatidylinositol
- RFP, red fluorescent protein
- *sst*, suppressors of sterility in *ste12*
- Ub, ubiquitin
- UBC, ubiquitin-conjugating enzymes
- UBPY, ubiquitin isopeptidase Y
- UEV, ubiquitin E2 variant
- UIM, ubiquitin-interacting motif
- VHS, Vps27p, Hrs and STAM
- Vps, vacuolar protein sorting
